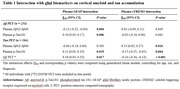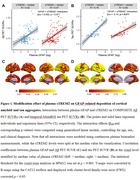# Blood‐based glial reactivity modulates cerebral amyloid and tau deposition in Alzheimer's disease

**DOI:** 10.1002/alz.088130

**Published:** 2025-01-09

**Authors:** Guoyu Lan, Anqi Li, Jie Yang, Laihong Zhang, Lili Fang, Yalin Zhu, Zhengbo He, Xin Zhou, Lin Liu, Pan Sun, Yue Cai, Tengfei Guo

**Affiliations:** ^1^ Institute of Biomedical Engineering, Shenzhen Bay Laboratory, Shenzhen, Guangdong China; ^2^ Institute of Biomedical Engineering, Peking University Shenzhen Graduate School, Shenzhen China

## Abstract

**Background:**

Plasma biomarkers of Alzheimer’s disease (AD)‐related pathological changes are considered as proxies of their changes in the brain. Recent cohort studies reported very early changes in plasma astrocytic marker glial fibrillary acidic protein (GFAP) in the AD spectrum, which is relative to the onset of neuropathology. Little is known about the association between plasma soluble triggering receptor expressed on myeloid cells 2 (sTREM2) and primary AD pathology. This study explored how GFAP and sTREM2 in plasma influence cortical β‐amyloid (Aβ) and tau deposition.

**Method:**

We measured the concentrations of plasma biomarkers by the Simoa or MSD platforms in a total of 285 individuals from the Greater‐Bay‐Area Healthy Aging Brain Study (GHABS) cohort, a large community‐based cohort study of older adults from China. All of them underwent Aβ PET imaging ([^18^F]‐D3FSP or [^18^F]‐florbetapir), and 104 individuals were additionally scanned for tau PET imaging. We investigated the modification effect of plasma glial biomarkers (GFAP and sTREM2) on Aβ PET and tau PET using generalized linear models, controlling for age, sex, and diagnosis.

**Result:**

Plasma GFAP but not sTREM2 interacted with Aβ_42_/Aβ_40_ and p‐Tau_181_ (Table 1), where higher GFAP levels were associated with steeper relations of lower Aβ_42_/Aβ_40_ and higher p‐Tau_181_ with elevated Aβ PET. Regarding tau PET, we found that higher plasma GFAP levels enhanced the association between plasma p‐Tau_181_ and tau PET. In contrast, higher plasma sTREM2 levels attenuated the associations of lower Aβ_42_/Aβ_40_ and higher p‐Tau_181_ with tau PET elevations. Congruent results were obtained when we used Aβ PET as the interaction term in the models. Furthermore, higher plasma sTREM2 levels also mitigated the association between plasma GFAP and tau PET but did not influence Aβ PET (Figure 1). At the voxel level, the association between plasma GFAP and tau PET was diminished and limited in individuals with above‐median sTREM2 levels versus individuals with below‐median sTREM2 levels.

**Conclusion:**

This study provides novel insights into the link between plasma glial biomarkers and cortical deposition of Aβ and tau aggregates, where plasma GFAP is linked to augmented Aβ and tau pathology, whereas plasma sTREM2 is linked to attenuated tau accumulation.